# Beluga whale summer habitat associations in the Nelson River estuary, western Hudson Bay, Canada

**DOI:** 10.1371/journal.pone.0181045

**Published:** 2017-08-02

**Authors:** Alexander J. Smith, Jeff W. Higdon, Pierre Richard, Jack Orr, Warren Bernhardt, Steven H. Ferguson

**Affiliations:** 1 Ramboll Environ, Tampa, Florida, United States of America; 2 Higdon Wildlife Consulting, Winnipeg, Manitoba, Canada; 3 Fisheries and Oceans Canada, Winnipeg, Manitoba, Canada; 4 North/South Consultants Inc. Aquatic Environment Specialists, Winnipeg, Manitoba, Canada; Macquarie University, AUSTRALIA

## Abstract

To understand beluga whale (*Delphinapterus leucas*) estuarine use in the Nelson River estuary, southwest Hudson Bay, we recorded and examined beluga movements and habitat associations for the July through August period in 2002–2005. We compared locations of belugas fitted with satellite transmitters (“tags”) (2002–2005) and aerial-surveyed (2003 and 2005) belugas for years of differing freshwater flow from the Nelson River which is influenced by hydroelectric activity. Using the beluga telemetry location data, we estimated an early August behavioral shift in beluga distribution patterns from local estuarine use to a progressively more migratory behavior away from the estuary. The timing of this shift in behavior was also apparent in results of beluga aerial surveys from the 1940s–1960s, despite environmental changes including later freeze-up and warming ocean temperatures. Overall, during the higher than average discharge (“wet”) year of 2005, the three tagged belugas ranged farther from the Nelson River but not farther from the nearest shore along southwestern Hudson Bay, compared to the 10 tagged belugas tracked during the “dry” years of 2002–2004 with below average discharges. Aerial survey data for 2003 and 2005 display a similar dry vs. wet year shift in spatial patterns, with no significant change in overall density of belugas within the study area. In the Nelson estuary, proximity to the fresh-salt water mixing area may be more important than the shallow waters of the upper estuary. Killer whales (*Orcinus orca*) were observed in the Churchill area (200 km northwest) during each year of study, 2002–05, and belugas may benefit from the proximity to shallow estuary waters that provide protection from the larger-bodied predator. Study results contribute to an understanding of the influence of environmental variation on how and why belugas use estuaries although considerable uncertainties exist and additional research is required.

## Introduction

Beluga whales (hereafter referred to as ‘belugas’, ‘beluga’; *Delphinapterus leucas* (Pallas, 1776)) tend to aggregate in estuaries in summer throughout their circumpolar range, which they occupy for several weeks to a few months. Beluga seasonal fidelity to estuaries potentially increases their sensitivity to environmental changes in those areas [1]. Climate change theory predicts that Arctic ecosystems will experience disproportionate impacts, with altered water levels and increasingly erratic weather patterns [2,3,4,5]. Past studies found that both water depth and weather affected the locations of belugas in estuaries [6,7,8]. Why beluga use estuaries is not well known, but hypotheses are numerous, and reasons likely vary geographically and across populations and may not be mutually exclusive. Hypotheses for beluga estuary-use include (1) feeding [9,10,11], (2) calving [12,13,14], (3) moulting [7,15,16], (4) avoiding killer whales (*Orcinus orca* (Linnaeus 1758)) [8,17], (5) avoiding humans [8,18], and (6) thermal advantage [12,19].

Western Hudson Bay beluga form one of three stocks in Hudson Bay and migrates seasonally through Hudson Strait to recurring summering areas in Hudson Bay [20]. From mid-June to October, western Hudson Bay belugas are distributed along the west coast of Hudson Bay forming large predictable aggregations within and near the Churchill, Seal, and Nelson River estuaries and adjacent coastal areas. Western Hudson Bay belugas are differentiated genetically from the neighboring Hudson Bay stocks and from more distant beluga populations [21]. The stock size of belugas using the estuaries in southern Hudson Bay (Churchill, Nelson, and Seal rivers) was estimated from an aerial survey in 1987 of 31,124 (SE = 6967) and in 2014 of 51,761 (15,875) both corrected for availability bias [22] and the stock is considered Special Concern [23]. In the Churchill River, Watts et al. [24] recorded beluga abundance with a sampling regime that included a temporal buffer around high tide, and previous studies in the Churchill River established a link between water temperature and beluga abundance [25].

We studied western Hudson Bay beluga habitat use of the Nelson River estuary related to artificially-altered freshwater flow down the Nelson River due primarily to the Limestone hydroelectric generating facility and other alterations of the water flow by Manitoba Hydro a Crown Corporation and the province of Manitoba’s major energy utility. Georeferenced locations of beluga were derived from animals instrumented with Argos^®^ satellite transmitters (hereafter referred to as “tags”) and from aerial surveys of the estuary area. If belugas prefer estuarine water in summer, then we predicted that greater numbers (density) and a larger range should occur farther out in the estuary during a year with greater freshwater flow due to the greater area of estuarine habitat. Alternatively, if belugas prefer the shallow water of the estuary then their distribution would not vary significantly between “wet” and “dry” years as they would continue to distribute close to the estuary shoreline that does not change with water discharge. We also estimate the timing change from local estuary use to autumn migration from 13 tagged belugas and compare results to historical estimates from aerial surveys. Using the time when tagged belugas left the estuary and migrated towards their winter range as a demarcation point, we defined local estuarine habitat as the area represented prior to migration. The study contributes to a better understanding of beluga summer habitat use and timing of their autumn migration, and may assist in habitat management of estuaries that are subject to anthropogenic changes, including hydroelectric activity.

## Materials and methods

### Study area

Arctic marine water, large inputs of fresh water, and nearly complete seasonal ice cover characterize Hudson Bay and together provide support for a complex Arctic marine food web far south of what is expected. In the main basin of Hudson Bay the bottom extends well offshore as a broad coastal shelf < 80 m deep and then slopes gradually to a smooth sea floor with an average depth of 250 m [26]. Seasonal ranges in surface temperature are relatively small with summer surface temperatures typically ranging from 1 to 9°C [26] Hudson Bay is essentially ice-covered in winter and ice-free in summer. Breakup begins in late May or early June along the coastline. The mammal fauna consists largely of migratory species that require access to air when ice is present and include belugas which are typically seasonal visitors to the region, although they overwinter in Hudson Strait and sometimes in leads and polynyas elsewhere [27]. The timing of their seasonal movements can vary by a month or so from year to year depending upon ice conditions. Hydroelectric developments, such as those on the Nelson, increase winter runoff [28] by storing water in large reservoirs for release later in the year. This has reduced the seasonal cycle resulting in a smaller spring freshet and increasing flow under the sea ice in winter [26]. The environmental impacts of shifting the seasonal runoff regime are not well understood.

### Determining “wet” and “dry” years

Mean daily discharge in cubic meters per second (m^3^/s) of freshwater flow from Limestone Dam (Manitoba Hydro, unpub. data) was averaged by year over the period of 14 July to 31 Aug, 1991–2006. Using the 16-year time series, each study year (2002–2005) was compared to the average flow rate and expressed as percent deviation from the average (S1 Table). Years with a greater than average flow rate were considered “wet”. Years with a less than or near average flow rate were considered “dry” but included years with average water discharge rate.

### Beluga capture and satellite telemetry

**Fig 1:**
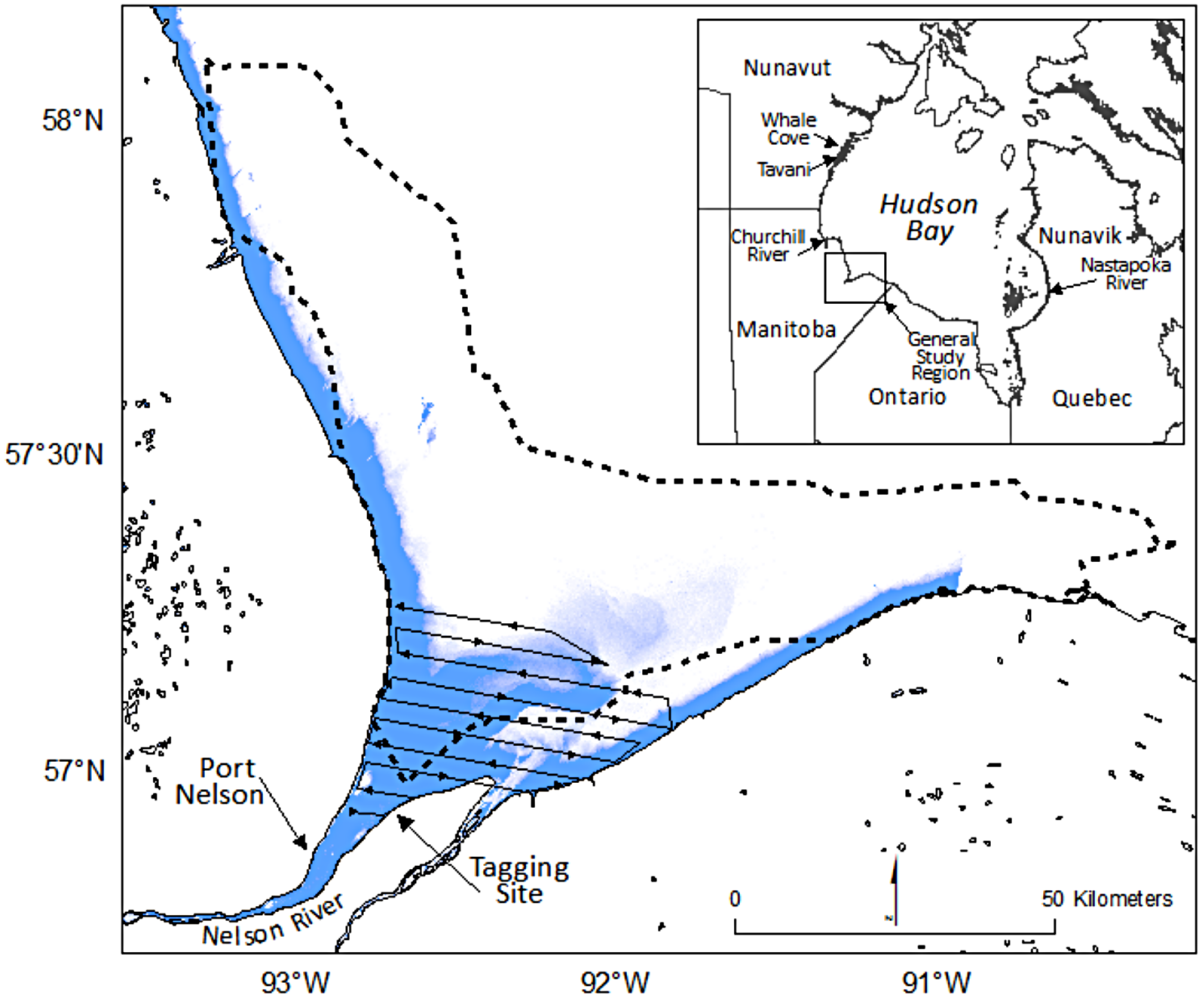
General study area (inset), including the Nelson River estuary in southwest Hudson Bay displaying the telemetry-derived estuary area (dashed line), the aerial survey transects, the site of beluga capture, and the freshwater plume (in blue) derived from a MODIS Aqua image from 8 August, 2000.

Thirteen belugas were instrumented with satellite tags in the Nelson River estuary, Canada (Fig 1) in late July or early August of 2002 to 2005 (Table 1). The belugas were captured and instrumented using a handling protocol approved by the Department of Fisheries and Oceans Canada Animal Care Committee and techniques described in Orr et al. [29]. Using a jet boat, the target beluga was encircled with a seine net in which it became entangled. Two inflatable boats approached the beluga and maintained it at the surface while moving it to shallower water, where it was removed from the net and secured with a rope around its tail and a hoop net over its head to restrain it for instrumentation. We attached Wildlife Computers^®^ model SPLASH and ST16 satellite transmitters (programmed identically) via wire cables to three polyethylene pins implanted through the skin and blubber layers of the dorsal ridge [29]. Sex, body length, and association with a calf were recorded. All calves were less than 3 years of age. The captured belugas included seven adult males, five adult females, and one sub-adult male. All captured females had calves. For three of the five adult females their calves (1–3 years of age) were also restrained, minimally handled, and released with their presumed mother. For the other two adult females, calves remained nearby while the female was being handled (ca. 30 min) but all were reunited upon release.

**Table 1:** Information on 13 belugas captured and instrumented with satellite tags at the Nelson River estuary in the summers of 2002–2005. Fc = female with calf (<3 years of age), M = male. Note that one of the male satellite-tagged belugas (2005: Tag No. = 57600) measured 265 cm and is considered a sub-adult.

Year	Tag No.	Wildlife Computers^®^ Tag Model	Deployment Date	Transmitter Longevity (# of days)	Sex	Length (cm)
2002	10927	ST16	13 July	21	Fc	320
2003	10899	ST16	5 August	83	M	370
	10926	ST16	4 August	112	Fc	375
	10971	ST16	30 July	107	M	405
	10972	ST16	3 August	117	Fc	344
2004	10978	ST16	24 July	224	M	410
	10979	ST16	24 July	268	M	400
	10980	ST16	25 July	231	Fc	340
	40622	ST16	26 July	71	Fc	408
	40623	ST16	27 July	201	M	409
2005	10970	Splash	23 July	37	M	330
	40153	Splash	30 July	50	M	310
	57600	Splash	30 July	213	M	265

Belugas were fitted with satellite transmitters and ARGOS System (www.argos.system.org) estimated the locations and provided a measure of the precision of those locations, or location quality classes [30]. Received ARGOS data for the 13 dive-recording tags were pre-processed using Wildlife Computers Inc. SATPAK software which chooses the best location solution from duplicate messages that occur in both ARGOS^®^ dispose (.ds) and diagnostic (.diag) raw file formats. Location data were then further filtered to only include "guaranteed” precision locations, (between 250 m and 1.5 km accuracy) with a travel rate of <3.5 km/hr. [31,32] retained 29% of the initial Argos dataset which was sufficient to minimize temporal autocorrelation.

### Determining timing of beluga migration

The Nelson Estuarine habitat was defined by assessing the temporal sequence of location data to determine the date at which there was a change between local movements and migratory movements. The change date was determined using a distribution free cumulative sign test [33]. The median of daily distances of tagged belugas to the river mouth (defined as the location of Port Nelson, 57.0552° N, 92.5967° W) were calculated. If a daily median distance was less than the overall median distance, a score of -1 was set for that day. When it exceeded the overall median distance, the score was set to +1. A cumulative sum of scores were calculated for each day and the absolute value of the cumulative sums was plotted to determine when the cumulative score was highest, indicating a change point in location from the river mouth (i.e., belugas median daily distances from the mouth of the estuary noticeably increased). The estuarine habitat was defined by beluga spatial distributions from satellite telemetry using Kernel Density methods [34] with the Animal Movement Analysis extension to ArcView 10.1 [35]. The Least Squares Cross Validation [36] smoothing parameters provided a less biased estimator than a user-selected or Worton's correction [34]. The fixed kernel home range utilization distributions were ESRI grids using LSCV including the recommended bivariate normal density kernel [34] and figures generated using the 95 probability percentage polygon.

### Aerial surveys

Seventeen high-tide aerial surveys were conducted in the Nelson River estuary during the summers of 2003 (n = 7) and 2005 (n = 10) (Table 2).

**Table 2:** Summary of 17 beluga aerial surveys conducted in the Nelson River estuary during high tides in 2003 and 2005.

	Date	Time	Tide
**2003**			
	02-Aug	1245–1430	Spring High
	03-Aug	1400–1600	Spring High
	04-Aug	1400–1600	Spring High
	06-Aug	1645–1845	Neap High
	09-Aug	0800–1000	Neap High
	12-Aug	1000–1215	Spring High
	13-Aug	1030–1245	Spring High
**2005**			
	29-Jul	1630–1830	Neap High
	30-Jul	1630–1845	Neap High
	31-Jul	1715–1930	Neap High
	01-Aug	1900–2045	Neap High
	03-Aug	0845–1100	Neap High
	05-Aug	1000–1215	Spring High
	07-Aug	1145–1345	Spring High
	10-Aug	1330–1515	Spring High
	11-Aug	1415–1615	Spring High
	13-Aug	1530–1730	Neap High

Systematic strip-transect surveys (100–450 m per side) were flown over the study area at an altitude of 305 m using a Cessna 337 Skymaster. Ten transects, oriented perpendicular to shore and extending up to 40 km offshore, were arranged ca. 3.7 km apart (Fig 1), a distance that limited the likelihood that belugas swimming perpendicular to the transects would be counted more than once during a survey. This provided direct sampling coverage of ca. 19% of the survey area. The extent of the surveys ranged ca. 70 km northeast of Port Nelson and included roughly half of the estuarine habitat area derived from telemetry data. The total count of belugas observed was summed for each 15-second interval that covered approximately 1 km of transect length and 350 m strip on each side of the aircraft. One observer on each side of the aircraft was instructed to restrict their view to less than 500 m and focus close to the track line. Data was treated as a strip census as coverage within the 100–450 m strip on each side of the aircraft was uniformly distributed [37]. Survey counts were treated as an index of density with no corrections for perception or availability bias. Weather covariates were not recorded.

### Spatial and temporal statistical analyses

Within the study area and time-frame, when the tagged belugas exhibited ‘local’ movements, their location distances to shoreline and the mouth of the estuary (response variables) were examined in relation to year, tide level [38], whale type (adult male or female with calf), year, and beluga identification (PTT). The funnel-shaped Nelson River estuary extends approximately 60 km offshore and is about 75 km along the coastline on each side. An adult beluga could cross the entire study area in a 24-hour period, we therefore used daily median locations rather than all locations to minimize spatial and temporal auto-correlation. The time series of ordinary least squares residuals were used to test for autocorrelation and partial-autocorrelation in the telemetry data and the choice of correlation structure (correlation = corARMA) fit to the process errors in R [39]. Likelihood-ratio tests (Durbin Watson) were used to confirm that autocorrelation was constrained in the model. Choice of model selected was guided by an information theoretic approach (AICs).

Two mixed-effects models were constructed in the statistical computing package R to compare differences in log (distance variables for tagged beluga locations) among exposure categories (Years with different water discharge) with random effect of individual tagged belugas (PTT). Habitat parameters, distance to nearest shoreline and distance to the river mouth or port (continuous), were treated as the dependent variables and we hypothesized these would be strongly influenced by water discharge. Additional covariates included year, day, tide level (m), sex class, and individual tagged beluga (PTT). Mixed-effects models using a Gamma distribution for continuous data were performed in R package gls, which allowed for the incorporation of an AR1 autocorrelation structure to account for the time-series nature of the observations [40]. Continuous predictor variables were screened for collinearity (Pearson’s correlation coefficient ≥ 0.6 or a variance inflation factor (VIF) > 3.0). Because the Nelson River estuary is a geographic area, distance to shore, distance to channel, and depth covaried (VIF) and so we avoided including all three covariates in the models. Since distance to shore was considered the more reliable measure and the river channel likely changes over time, we chose to include only distance to mouth of the estuary and distance to the shoreline as the spatial covariates in the model.

For the aerial survey data, survey transects were divided into 1 km by 0.7 km. blocks (ca. 700 m^2^) representing sampling units. Density was compared between 2003 and 2005 using aa generalized linear model (semi Poisson with a log-link function) with explanatory variables: year, survey, distance to shore, and distance to mouth to assess possible differences in beluga density between the normal and wet years. Akaike Information Criterion for small sample sizes (AICc) was used to assist with model selection, where the lowest relative ΔAICc was used to select the most parsimonious model using MuMIn v1.15.1 [41] in R.

Next, to assess possible spatial re-distribution of belugas with water discharge as the treatment, we weighted the distance values by density (counts within a survey block) and used mixed effects models (Gamma distribution) to test for year effects while controlling for the random effect of individual surveys (day of survey). Unlike the telemetry data, the aerial surveys included one dry year (2003), thus eliminating the requirement for cross-year data pooling. The surveys covered the study area in less than 2 h and the repeated surveys were controlled for by date, thus we assumed that density of survey blocks were independent. We made no direct comparison between the telemetry and aerial survey data due to the different spatial and temporal scales of the two methods.

## Results

### Defining spatial and temporal estuary use

**Fig 2:**
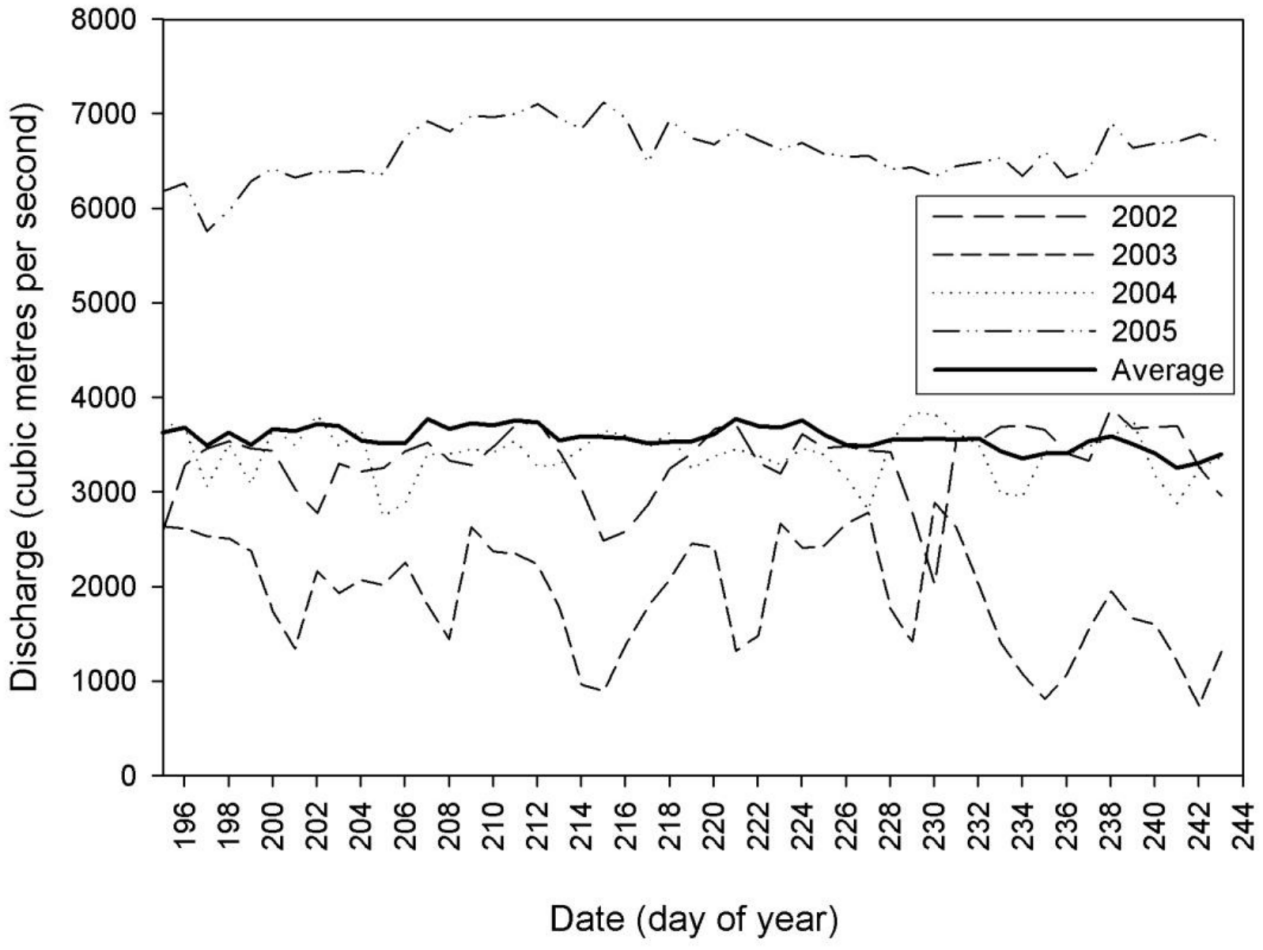
Comparison of Nelson River water discharge (cubic meters per second) for 2002–05 from 14 July (196) to 31 August (244) when belugas inhabit the Nelson River estuary. Solid dark line is average daily discharge, 1991–2006, which was used to define "wet" (2005) and "dry" (2002–04) years in comparing beluga distribution.

The average freshwater flow rates from Limestone Dam for the period of 14 July to 31 August in 1991–2006 ranged from 2167 m^3^/s in 2004 to 5176 m^3^/s in 2005 with an average flow rate of 3331 m^3^/s (S1 Table). The percent deviations from the overall average flow rate for each of our study years (2002–2005) were -6%, -35%, -15%, and +55%. Thus, 2002–2004 were “dry” years and 2005 was a “wet” year. Flow rates for 2002–2004 pooled were 19% drier than the average from the 16 year time series. There was an 83% increase in flow rate from 2004 to 2005 (Fig 2).

### Do belugas re-distribute across river discharge years

**Fig 3:**
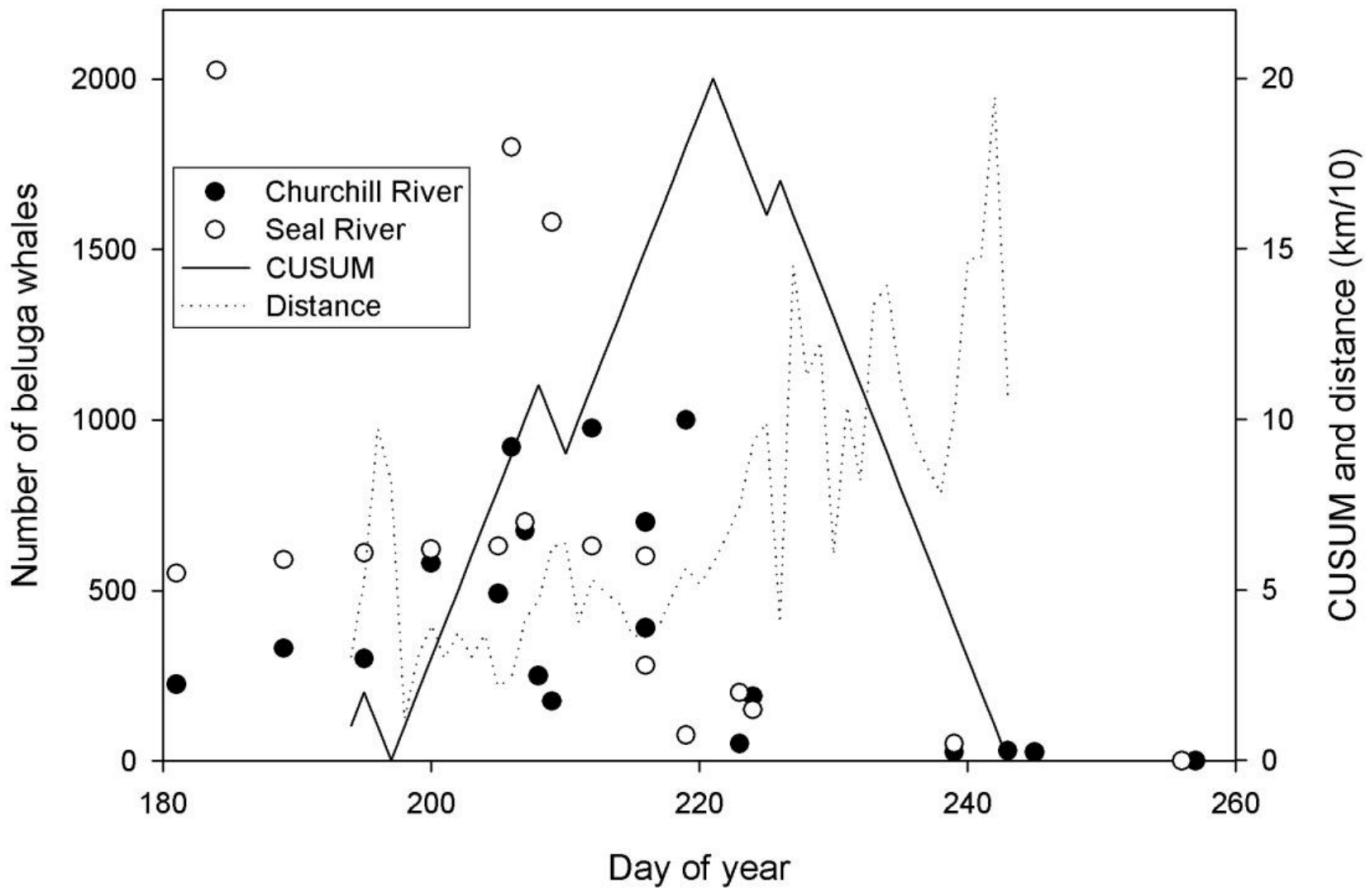
Results of change point test method of 13 satellite tagged beluga from 2002–2005 to demarcate the movement shift from local estuary use to autumn migration (9 August = day 221). The solid line is the change point test result that uses mean daily-pooled distance from the Nelson River mouth (dashed line). Historical aerial survey sightings for the Seal and Churchill Rivers estimated from a figure in Sergeant [12] for illustrative purposes displaying that the timing of the decline in numbers of belugas sighted in the Seal and Churchill rivers coincides with change-point test results for a behavioral shift in beluga movements in the Nelson River estuary.

To determine habitat associations of tracked belugas, we needed to consider that although transmitter longevity differed for each beluga (Table 1), they lasted through the end of the seasonal study period for all animals except one. Tag 10927 had the shortest duration of the 21 tags and stopped transmitting on 3 August 2002. The absolute cumulative sign value for the pooled distance data reached a maximum on calendar day 221, which corresponds to 9 August (or 8 August for 2004, a leap year). This suggests a behavioral shift from local estuarine occupation to more migratory movements around that date. Boundaries of the estuarine habitat were obtained by bounding pre-9 August beluga telemetry location data (Fig 3). This estuarine habitat includes 150 km of shoreline and extends approximately 60 km offshore from the entrance of the Nelson River (S2 Table). Belugas used the estuarine habitat through 9 September but with successively fewer beluga after the second week of August.

**Fig 4:**
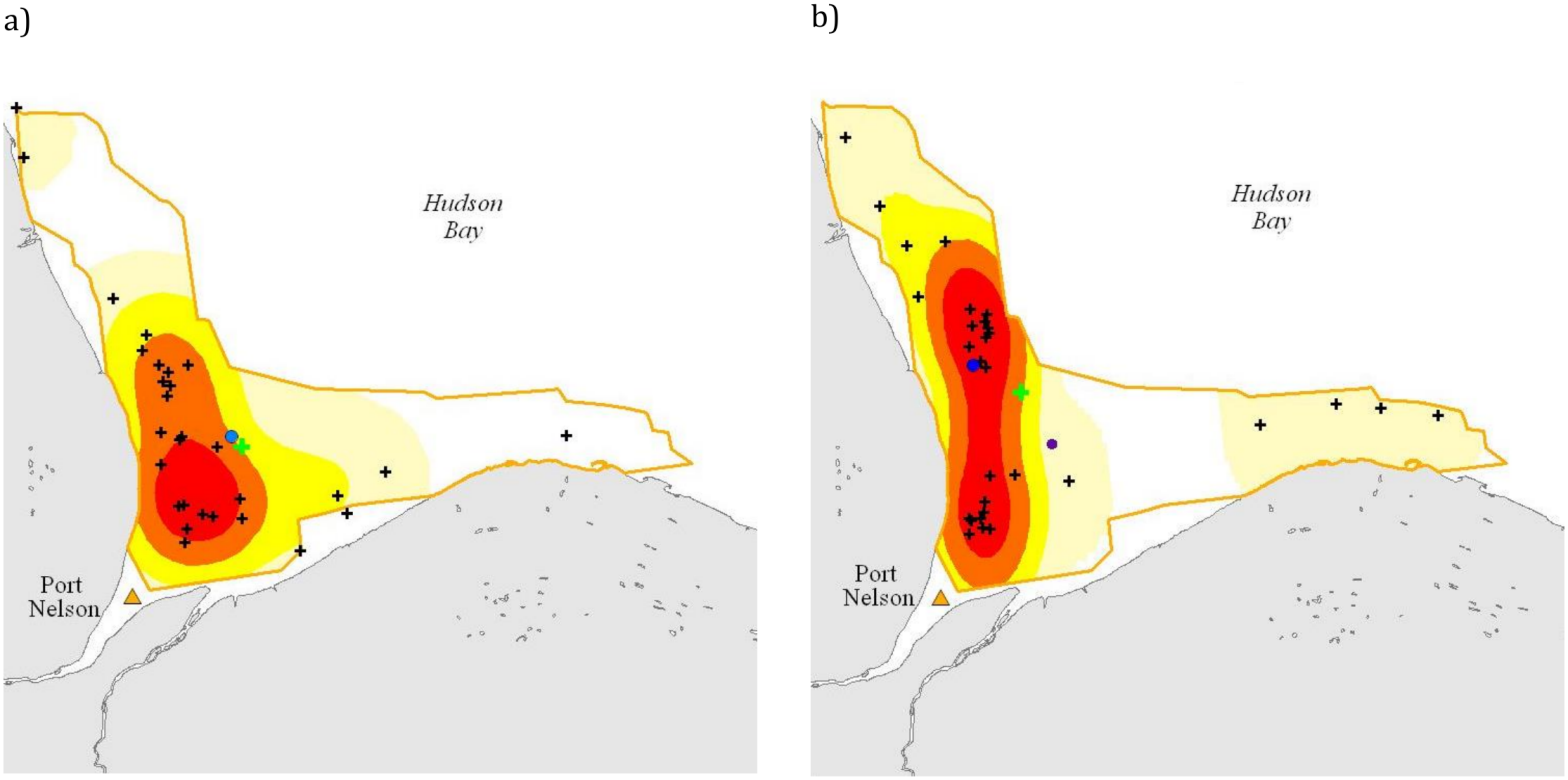
Nelson River beluga utilization distributions based on Kernel probability density estimation (PDE) analyses of satellite tag locations for the drier years of 2002–2004 (a) and for the wet year of 2005 (b). Utilization distributions are displayed as red < = %25, orange < = 50%, yellow < = 75%, pale yellow < = 95%. The green cross = the geometric mean (central tendency using the product of values as opposed to the arithmetic mean which uses the sum) location of all tagged belugas, blue dot = the center of mass (distribution where the weighted relative position of the locations sums to zero) for the 95^th^ percentile Kernel utilization distribution, orange triangle = Port Nelson, black crosses = median daily locations for each beluga.

Results of the mixed-effects models indicated that distribution of tagged belugas differed between 2005, the wet year, when the central location was approximately 12 km farther from the Nelson River mouth than in the drier years of 2002–2004 (Fig 4). The information theoretic (AIC corrected for small sample size) approach indicated that for Distance to shore the best models included Dry/Wet year difference, Sex, and individual beluga; whereas for Distance to mouth the best models included Dry/Wet year differences, Tide, Sex and individual beluga (Table 3). Distance to shore was greater during dry (14.0 km median (9.1–19.6 25^th^ and 75^th^ percentiles), n = 511) versus wet years (12.1 (7.7–16.2), n = 915); whereas tagged belugas were located farther from the mouth of the estuary during the wet year (63.3 km, (48.8–91.5), n = 915 versus 50.2 (35.9–68.8), n = 511). Relative to wet and dry years, beluga distribution differed significantly with respect to tide level with greater use of the estuary during high tide (Table 3). Time of day did not significantly affect distance to river mouth or distance to nearest shore. Males and females did not differ in distance to the river mouth (56.4 km median (39.0–202.3 25^th^ and 75^th^ percentiles), n = 8 versus 58.9 km (42.1–81.8), n = 4 females) or in distance to shore (13.3 km (8.7–18.6) versus 12.6 (8.1–17.2)).

**Table 3:** (A) Two sets of generalized mixed-effects model with autocorrelation (AR1) of 2002–2005 Nelson River estuary satellite-tagged beluga. Combinations of a number of explanatory variables were tested for differences in Log (distance to river mouth) and log (distance to nearest shore). Explanatory variables included year (Dry versus Wet), tide level, and sex class (males or females with calves) with random effect of individual beluga (PTT). (B) Mixed effects model fits predicting distance from shore (Dsh) of tagged beluga.

(A)					
Dependent Variable (km)	Coefficients	Estimate	Standard Error	t value	p-value
Log (Distance to river mouth)	Intercept	2.825	0.1308	21.59	<0.001
	Dry/Wet	-0.0694	0.08926	-0.7774	0.437
	Beluga	-0.0000124	0.00000207	-6.0048	<0.001
	Sex	0.1789	0.1167	1.5328	0.126
	Tide	-0.06741	0.01384	-4.8696	<0.001
Log (Distance to shoreline)	Intercept	11.2819	1.40677	8.01969	<0.001
	Dry/Wet	-3.025040	0.3632439	-8.327849	<0.001
	Beluga	-0.000070	0.0000068	-8.327849	<0.001
	Sex	-0.048191	0.1543758	-0.312169	0.755
	Tide	-0.027618	0.0057225	-4.826192	<0.001
(B)					
Model log(Dsh) ~	Df	LogLik	AICc	Delta	Weight
(Int) + Tide +Sex + PTT	5	-1682.236	3374.5	0.00	0.459
(Int) + Dry/Wet +Sex + Tide + PTT	6	-1681.679	3375.4	0.90	0.293
(Int) + Tide + PTT	4	-1684.174	3376.4	1.86	0.181
(Int) + Tide + Dry/Wet + PTT	5	-1684.167	3376.4	3.86	0.067
(Int) + Sex + PTT	4	-1690.346	3388.7	14.21	0.000
Model log(Dmo) ~	Df	LogLik	AICc	Delta	Weight
(Int) + Dry/Wet + Tide +Sex + PTT	7	-346.450	707.0	0.00	0.721
(Int) + Dry/Wet + Tide + PTT	6	-348.408	708.9	1.90	0.279
(Int) + Dry/Wet + Sex + PTT	6	-358.625	729.3	22,33	0.000
(Int) + Dry/Wet + PTT	5	-360.315	730.7	23.70	0.000
(Int) + Sex + Tide + PTT	6	-369.083	750.2	43.25	0.000

Akaike Information Criterion (AICc) is relative to the best-fitting model and weights calculated from likelihood ratios are relative to the best model in the set of models. (Int) is intercept, Wet-Dry is 2002–2004 versus 2005, Tide, Sex (male or female with calf), and random beluga variable (PTT) for parameter estimates of selected models.

**Fig 5:**
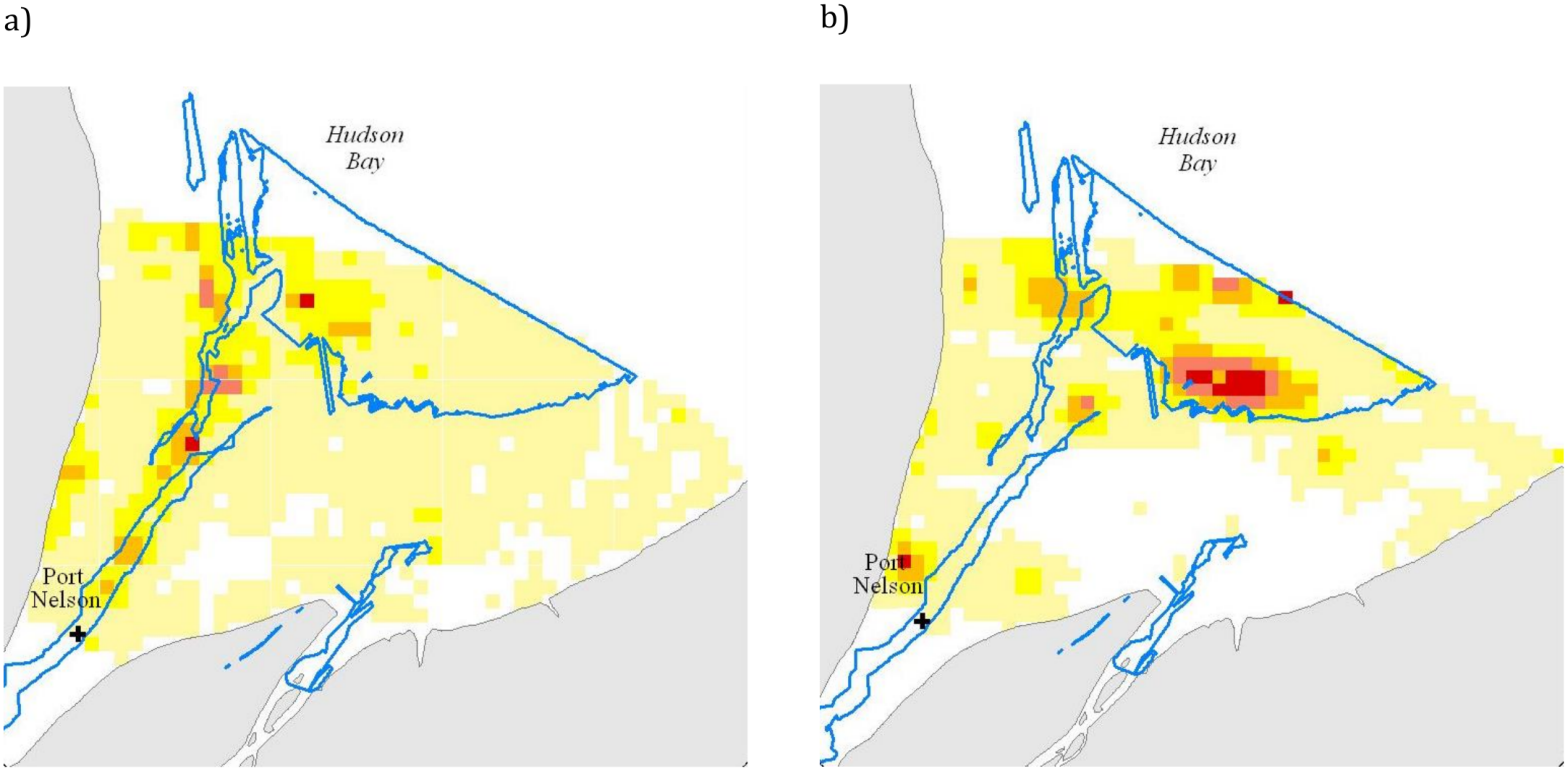
Aerial survey beluga density (km^2^) for (a) dry year 2003 and (b) wet year 2005. Density (km^2^): red = >40%, orange = 20–40%, yellow = 10–20%, pale yellow < = 10%. The blue line represents the channel.

Next, we determined habitat associations of aerial-surveyed belugas using generalized linear mixed model results (S3 Table). The best model include Distance to the river mouth, Dry/Wet years, and survey (Table 4). During the wet year versus the dry year, beluga density was greater (2005; 13.7 + 0.38/km2, n = 1764 versus 2003; 11.3 + 0.26, n = 2183), belugas were farther from the river mouth (26.9 ± 1.38 versus 25.8 ± 1.21 km) and closer to shore (8.65 ± 0.139 versus 10.25 ± 0.121 km; Table 4b). Beluga density was greater farther out in the estuary in the wet year (2005) but due to the funnel shape of the estuary they were similarly concentrated relative to shore during the dry year (2003) (Fig 5). Compared to 2003, beluga density was higher along the western shore channel. More belugas used the survey area in 2005 (mean = 9,355, n = 7 survey replicates) compared to 2003 (mean = 7,365, n = 10 survey replicates).

**Table 4:** Generalized mixed model of Nelson River estuary beluga aerial surveys testing for differences in distance to shoreline and distance river mouth (weighted by beluga density) with 2003 (normal) and 2005 (wet) years and controlling for survey date as random effect.

Dependent Variable (km)	Coefficients	Estimate	Standard Error	t value	p-value
Weighted	Intercept	-1.135e+06	3.833e+05	-2.960	0.0031
distance	Dry/Wet	5.887e+02	1.918e+02	3.069	0.0022
to river	Survey	-8.700e+01	3.633e+01	-2.395	0.0167
mouth					
Weighted	Intercept	571420.57	192275.44	2.972	0.0030
distance	Dry/Wet	-279.29	96.21	-2.903	0.0037
to	Survey	-11.30	18.22	-0.620	0.535
shoreline					

**Fig 6:**
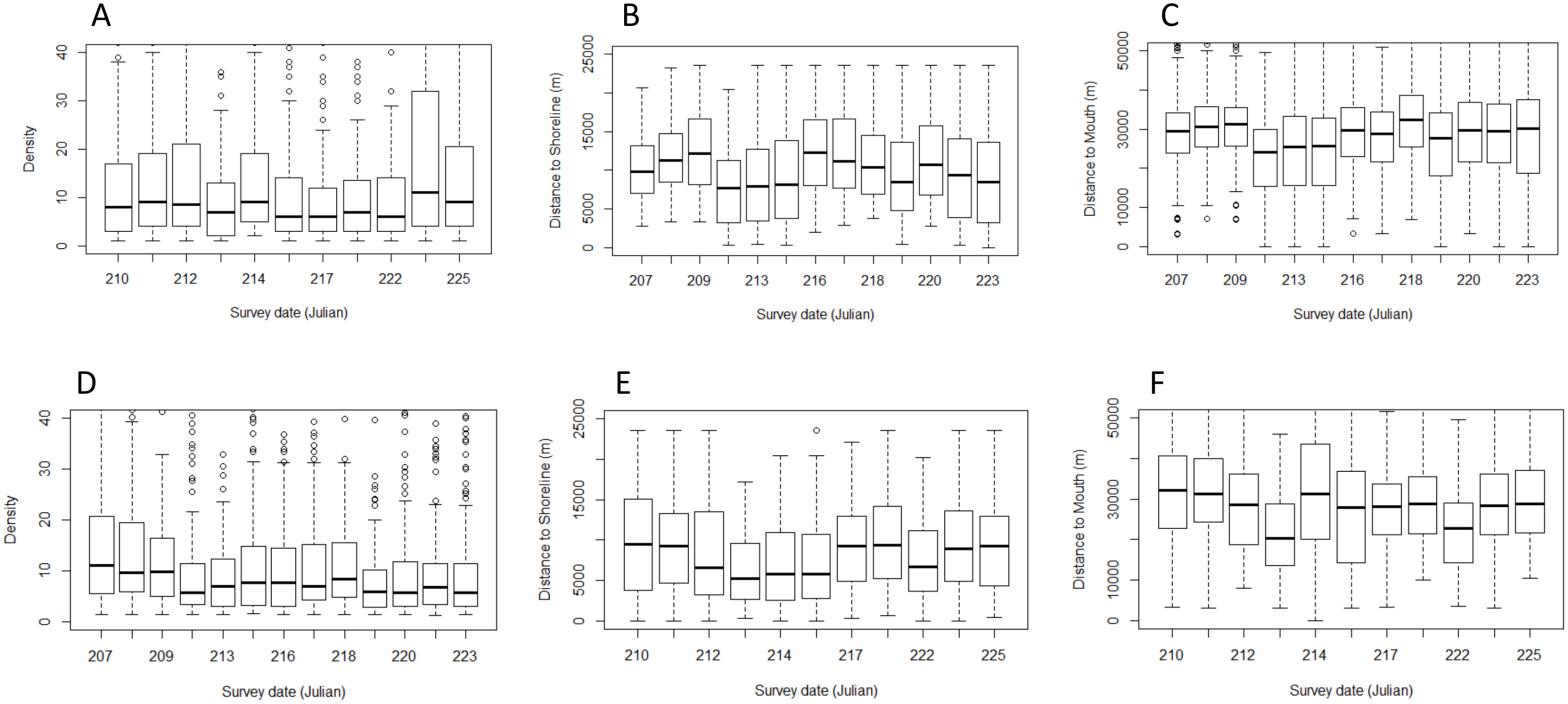
Mean density of belugas (a, d), distance to shore (b, e), and distance to river mouth (c, f) as a function of Julian day of aerial survey during the dry year of 2003 (top row) versus the wet year of 2005 (lower row).

Beluga density and distribution relative to shore and the river mouth varied seasonally with an overall higher density recorded in the wet year(Fig 6). Belugas may have been closer to shore and river mouth during the 30 July (211 Julian day) to 3 August (215) period during both 2003 and 2005 (Fig 6). Over the study period, 2002–05, killer whales were observed in the Churchill area three times in 2002 (late August), once in July-August in 2003, once on August 1st 2004, and twice during the summer of 2005 (no dates provided; DFO data on file).

## Discussion

During the wet year, beluga distribution determined by telemetry was generally farther from the river mouth in slightly deeper, perhaps more saline water, but still within the estuary proper. In comparison, during the dry year beluga were closer to the river mouth while remaining relatively close to shore. Although we did not measure salinity, a possible explanation for the change in distribution was that beluga concentrated near the freshwater and saltwater mixing area that varied in location depending on the volume of water discharge. Aerial survey results were similar to telemetry data, with beluga densities in 2005 slightly higher between 23 km from the river mouth and the aerial survey offshore extent (66 km) than during 2003 surveys. The smaller spatial extent of the aerial surveys, relative to the area used by tagged animals, limits the comparison of the two data sets. In addition, the aerial surveys ended by 13 August limiting the opportunity to use this data set to test when belugas started their autumn migration. The telemetry data indicated that some belugas were located farther north (offshore) than the limit of the aerial surveys. Interestingly, aerial surveys recorded belugas upstream of Port Nelson but none of the tagged belugas were recorded there. This likely relates to the limits of our telemetry sample size; 13 belugas from approximately 37,100 in the Nelson River estuary stratum [22]. However, we recognize that caution should be associated with lack of telemetry locations in fresh water as [42] have suggested that the lack of telemetry positions may be due to the tag capabilities themselves and the use of a saltwater switch.

### Defining spatial and temporal estuary use

The Nelson estuarine area used by belugas was delineated by bounding pre-August 9 tag locations with a polygon. This is a conservative approach to habitat use delineation since convex polygons or other similar methods would include regions outside of recorded animal locations, possibly overstating the estuary area. We used a change point test to define migration timing and this technique may be applicable to other migrating species. Future research would benefit from using state space models to test whether change point analysis results in similar temporal findings indicating a behavioral shift from lingering to moving [43]. Our analyses indicated that tagged beluga locations were influenced by tide and potentially by weather and other disturbances, but time of day was not significant. A significant relationship, however, was found between tide and distance to river-mouth suggesting that fresh water outflow from the Nelson River influences beluga distribution.

Our analysis identified the second week of August as the time that tagged belugas changed from local estuarine occupation to more migratory-like behavior. Sergeant [12] plotted beluga numbers observed by date for aerial surveys of the Seal River and Churchill River estuaries conducted from 1948 to 1967 (all years combined). The decline in number of belugas counted after the first week of August is similar to our results of Nelson River estuary increasing daily-distance of tagged belugas from the Nelson River after early-August. These results suggest that the timing of intensive estuary use and movement out of these estuaries, have remained largely unchanged since the 1950-1960s (Fig 3), despite environmental effects of climate change [44]. Matrilineal behavior learning may have ‘‘locked” these belugas into traditional habitat use and consequently may constrain their behavioral plasticity to environmental change [45].

### Why use estuaries

Different beluga populations may enter estuaries for different reasons that may not be mutually exclusive and may vary geographically and/or between populations. In Cook Inlet, Alaska, no clear relationship is apparent between beluga distribution and any one factor; however, tide, water depth, and temperature may influence beluga distribution near river deltas [46,47]. More research is needed to relate spawning fish runs up rivers and beluga feeding in river mouths. In Russian waters during high spring tides, prey availability presumably motivates the coastal movements of belugas into rivers [10,48,49]. In the Nastapoka River, eastern Hudson Bay, beluga distribution and behavior were influenced mainly by tide and total number of belugas present [50]. The median position of the beluga group advanced and retreated with the flow and ebb of the tide. Length of the period without human disturbance, high waves, strong northerly winds, high river water temperature, and clear water all favored the occupation of the upper reaches of the estuary [8]. In Cunningham Inlet, twice daily during low tide, belugas dispersed along the outer edge of the foreshore area [7]. Feeding was probably not involved since the muddy tidal flats contained little benthic life, and their behavior was described as loafing. In the eastern Beaufort Sea, water temperature appeared to influence beluga locations in the Mackenzie estuary while water depth, salinity, turbidity, and shelter were deemed less important [14,19].

The proximate reasons for beluga estuarine fidelity remain difficult to define and the importance of estuaries in beluga life history has largely been inferred from their continued occupation despite disturbances [7,51,52]. Anthropogenic disturbances, however, may cause progressive wariness in belugas and increasingly longer post-disturbance abandonment of estuaries [6,52]. Belugas move frequently between salt and fresh water [53] and seasonal occupation of freshwater habitat does not appear to be a necessary condition for beluga survival [54]. Calving has been suggested as a primary reason for beluga estuarine aggregations in summer [11,12,24]. There is little evidence of reproductive activity in estuaries. The estimated mating season occurs earlier in the spring [12,17,55]. A related explanation is that belugas use the estuaries following parturition as nursing areas for neonates. However, our telemetry results indicate that females accompanied by neonates (n = 4) were generally slightly closer to the river mouth relative to males (56.4 versus 59.0 km) but close to the shoreline (13.3 km) where presumably safety is available in shallow waters where killer whales cannot go due to their larger body mass. Unfortunately, the alternative hypotheses for why belugas locate within estuaries in summer are not mutually exclusive.

Belugas in the Nelson River estuary proper and those occurring offshore were in relatively close proximity to the shallow estuarine habitat that may provide escape options from predators. In the wet year of 2005, the tagged belugas travelled farther up the coast, north of the estuary, but still remained within 15 km of the shoreline where the water is shallow, which suggests a coastal preference. They may prefer access to shallow water but very shallow water also carries a risk of stranding with the tide [56]. Killer whales have been seen pursuing and eating belugas as they escape to shallow water where they became susceptible to human hunters or stranding by the ebbing tide [6,57,58,59]. Predation modelling estimated that killer whales in Hudson Bay could remove on average of 174 belugas (range 12–326) annually [58] so risk of predation is plausible. If belugas seek protection from killer whales by using the shallow waters of the Nelson River estuary then a shift of beluga locations closer to the shallower river mouth is expected during years with higher water levels in order for the belugas to remain in the relative shelter of shallow water. This behavior was not observed during our study: however killer whales were reported sighted in the general study area (Rankin Inlet to Nelson River) four times in 2002, no observations in 2003, once in 2004, and seven times in 2005, possibly the same group re-observed multiple times each summer [60].

Foraging could be a primary reason for estuary use, but no foraging studies have been conducted for belugas in the Nelson River estuary to test this suggestion. Farther north in the Churchill River estuary, feeding occurs, mainly on capelin (*Mallotus villosus* (Müller 1776)), but does not appear to be considerable [61]. In Whale Cove, Nunavut, north of Churchill, late summer feeding may be more important [12]. Kelley et al. [62] found biomarker evidence suggesting capelin is an important food source for Western Hudson Bay belugas. Similarly, Doan and Douglas [6] observed vast schools of capelin along the shore of Hudson Bay at Churchill yet they were infrequently seen in the stomachs of belugas caught in the estuaries. Sergeant [12] recorded capelin in only 7% of stomachs examined in 1955 from the Churchill estuary. Fraker et al. [19] found that most belugas harvested by Inuvialuit hunters in the Mackenzie estuary, NWT had empty stomachs and suggested that food availability was not a major factor for beluga estuarine concentrations. Belugas in Cook Inlet, Alaska may be primarily attracted to the area by returning salmon in spring and summer, although their specific diet remains restricted to a small seasonal sample [63]. Their blubber thickness appears to coincide with extensive summer feeding followed by a reduction in prey availability in winter [48]. In Clearwater Fiord, Cumberland Sound, Nunavut, belugas stomachs are often empty and individuals leaving Clearwater Fiord in the fall are noticeably thinner, apparently due to a summer diet shift to opportunistic feeding on invertebrates while their fall and winter feeding targets pelagic and benthic fishes [18,64,65].

Belugas may occupy estuaries for thermal advantage and warmer estuarine waters may be important to all segments of a beluga population, not just for mothers and calves [13,17,19,21]. Inuit hunters have also suggested that warmer water provides the primary beluga estuarine habitat [7]. Belugas tagged in the Nelson River estuary were primarily found in the warmer freshwater. The large beluga groups observed in our aerial surveys beyond the salt-freshwater mixing zone may also intermittently take advantage of the warmer freshwater in close proximity. Many other cetacean species also migrate *en masse* to warmer calving waters [66,67].

Belugas, unlike other cetaceans, appear to undergo a seasonal moult that coincides with their estuarine occupancy [15,25,68]. Like some pinnipeds, belugas may benefit when elevated temperatures coincide with a seasonal moult [25,69]. Not all belugas reach estuaries each year, however, and some may remain in offshore waters for most of the summer [55,69,70]. Lower oceanic temperatures apparently do not metabolically stress newborn calves [20,24]. The skin of most of the 13 tagged belugas in the Nelson River appeared yellowish and rubbing on the rough bottom substrate of the estuary may help to remove old yellowing epidermis and may explain their site fidelity [7,24,51]. The locations of tagged belugas in the mixing zone of the Nelson River estuary could be explained by their intent to regulate their exposure to fresh and/or warmer water and thus the rate of epidermal proliferation.

Studying belugas in the large Nelson River estuary is a challenge but our spatially- and temporally limited results suggest that beluga in this region have re-distributed as a result of modifications to the river system by hydroelectric activity and changes to seasonal water discharge. Past studies raised concerns about potential negative effects of hydroelectric activity to belugas [23,37,71,72,73]. Woodley and Lavigne [72] suggested that alterations of temperature and salinity might make estuaries unsuitable for beluga moulting. Conversely, reduced flow from hydroelectric development of the Churchill River, 200 km to the northwest of the Nelson, did not result in a decrease in the number of belugas using the Churchill River estuary, despite earlier concerns [71]. The strengths of this study lie in the comparison of the data from two independent methods of observation, satellite telemetry and aerial surveys [74]. The aerial survey data supported the satellite telemetry data. Telemetry data also allowed us to determine the timing of change from beluga occupation of the estuary to more migratory-like behavior, and to delineate the boundaries of habitat use during the summer resident period. Estuarine use is a common feature of many beluga populations and further research is necessary to understand beluga behavior and reasons for fidelity to estuaries in summer. Such knowledge is critical in order to assess the ability of belugas to adapt to environmental change and human industrial development activities of their estuarine habitats.

## Supporting information

S1 Table Mean daily Nelson River water discharge records in cubic meters per second (m3/s) of freshwater flow from Limestone Dam (Manitoba Hydro, unpub. data) averaged by year over the period of 14 July to 31 Aug for 1991–2006. (XLS)

S2 Table Environmental information distance to mouth of estuary and distance to shoreline for 13 belugas captured and instrumented with satellite tags at the Nelson River estuary in the summers of 2002–2005. (XLSX)

S3 Table Environmental measures summary from 17 beluga aerial surveys conducted in the Nelson River estuary area during high tides in 2003 and 2005. (XLSX)
